# The Impact of Admission Serum Creatinine Derived Estimated Glomerular Filtration Rate on Major Adverse Cardiac Events in ST-Segment Elevation Myocardial Infarction Patients Undergoing Primary Percutaneous Coronary Intervention

**DOI:** 10.14740/jocmr2482w

**Published:** 2016-02-27

**Authors:** Mahmut Uluganyan, Gurkan Karaca, Turker Kemal Ulutas, Ahmet Ekmekci, Eyup Tusun, Ahmet Murat, Bayram Koroglu, Huseyin Uyarel, Nijad Bakhshaliyev, Mehmet Eren

**Affiliations:** a Clinic of Cardiology, Kadirli Government Hospital, Osmaniye, Turkey; b Clinic of Cardiology, Osmancik Government Hospital, Corum, Turkey; c Clinic of Biochemistry, Kadirli Government Hospital, Osmaniye, Turkey; d Clinic of Cardiology, Dr Siyami Ersek Thoracic and Cardiovascular Surgery Center Training and Research Hospital, Istanbul, Turkey; e Department of Cardiology, Bezmialem Vakif University Medical Hospital, Istanbul, Turkey

**Keywords:** Estimated glomerular filtration rate, Cockcroft-Gault, Mortality, ST-segment elevation myocardial infarction, Major adverse cardiac events

## Abstract

**Background:**

The impact of Cockroft-Gault (C-G) derived estimated glomerular filtration rate (eGFR) on mortality and major adverse cardiac events (MACEs) in patients with ST-segment elevation myocardial infarction (STEMI) undergoing primary percutaneous coronary intervention (PCI) was assessed.

**Methods:**

A total of 884 patients were classified into four categories according to admission creatine derived eGFR: < 60, 60 - < 90, 90 - < 120, and ≥ 120 mL/min/1.73 m^2^.

**Results:**

In-hospital and long-term MACEs were significantly higher in eGFR < 60 mL/min/1.73 m^2^ subgroup (P < 0.001 and P = 0.028). Multivariate analysis demonstrated 7.78-fold (95% CI: 0.91 - 66.8) higher mortality risk in eGFR < 60 mL/min/1.73 m^2^ subgroup.

**Conclusion:**

As an easily applicable bedside method, C-G derived eGFR could be important for prediction of in-hospital and long-term mortality and MACE in STEMI patients undergoing primary PCI.

## Introduction

Chronic kidney disease (CKD) is a significant and independent cause of cardiovascular adverse events and mortality in general population and patients with cardiovascular diseases [1-4]. Additionally, in many studies, it has shown that any degree of renal impairment assessed by estimated glomerular filtration rate (eGFR) is related to increased adverse cardiovascular outcomes and predicts in-hospital, short- and long-term mortality in acute coronary syndrome (ACS) and general population [5-8]. Decreased renal function assessed by eGFR predicts prognosis in both genders in ST-segment elevation myocardial infarction (STEMI) [9]. In STEMI patients undergoing primary percutaneous coronary intervention (PCI), poor myocardial perfusion and increased no-reflow phenomenon were detected in patients with decreased eGFR [10, 11]. Two recent studies have shown relation between decreased eGFR and increased burden of atherosclerosis and decreased left ventricular ejection fraction (EF) in various forms of ACSs [12, 13]. Renal dysfunction is associated with female gender, older age, hypertension (HT), and diabetes mellitus (DM) [7]. These factors are linked with worse clinical outcomes.

In the present study, we investigated the relation between admission serum creatine (Cr) derived eGFR and in-hospital and long-term adverse cardiac events in STEMI patients undergoing primary PCI.

## Materials and Methods

This retrospective observational single-centered study was conducted between May 2009 and 2011. Patients presenting with STEMI undergoing primary PCI were enrolled. Patients with unstable angina, non-STEMI and STEMI that did not undergo primary PCI were excluded. The European Society Cardiology/American College of Cardiology Foundation/American Heart Association (ESC/ACCF/AHA) committee advisement of ECG criteria was used for diagnosis of STEMI [14, 15]. These were definitive new bundle branch block or > 0.1 mV new ST elevation in two contiguous leads. The procedures were conducted by a highly experienced staff (performing > 75 PCIs/year), in a high volume center (> 3,000 PCIs/year).

Following the diagnosis of STEMI, the blood samples were obtained from the patients at the emergency department. The eGFR was calculated from these initial blood samples.

Cockroft-Gault (C-G) equation: C-G = ((140 - age) × weight (kg))/((72 × creatinine) × (0.85 if female)) was used for eGFR [16]. Patients were classified into four categories according to the eGFR (< 60, 60 - < 90, 90 - < 120, and ≥ 120 mL/min/1.73 m^2^).

Baseline demographic data were collected from the medical records. The left ventricular EF was measured just before discharge. The modified Simpson’s method was used for measurement via a System V (Vingmed, GE, Horten, Norway) [17].

The written informed consent was obtained from all patients and hospital local ethic committee approved the study protocol.

### Coronary angiography and primary PCI

All coronary angiographies and primary PCIs were performed via the femoral approach. The infarct related artery was graded by using thrombolysis in myocardial infarction (TIMI) classification [18]. The coronary artery stenosis was assumed as significant if stenosis was > 50% of diameter. The primary PCI was applied just to the infarct related artery. Following the diagnosis of STEMI, prior to primary PCI, all patients were loaded with 300 mg clopidogrel and 300 mg aspirin (unless contraindicated). Due to payment strategy of the Minister of Health, ticagrelor and prasugrel were not used. No statin loading was applied before primary PCI. After the revealing of the coronary anatomy, all patients were administered 100 U/kg heparin. Following the procedure, patients were transferred to the coronary care unit. Intravenous heparin (500 U/h) or subcutaneous low molecular heparin (enoxaparin) (1 mg/kg/day) was maintained in coronary care unit. Certain medications, e.g. aspirin 100 mg/day, clopidogrel 75 mg/day, rosuvastatin 20 mg/day or atorvastatin 40 mg/day, angiotensin converting enzyme inhibitor or angiotensin receptor blocker, and beta-blockers were administered according to the hemodynamic status of the patients. Glycoprotein IIb/IIIa receptor blocker (e.g. tirofiban) was used according to the burden of the thrombus on visual assessment.

### Definitions

The Killip clinical examination was used for definition of patient clinical condition at presentation. HT was defined if a patient was on an antihypertensive treatment or requiring an antihypertensive drug in hospital. Any patient on an oral anti-diabetic drug and/or insulin was assumed to be diabetic. Major adverse cardiac events (MACEs) consisted of cardiovascular mortality, re-infarction and target vessel revascularization (TVR). Re-infarction was defined by ST-segment elevation along with at least two-fold increase in serum creatine kinase-myocardial band (CK-MB) fraction activity.

### Statistical analysis

All quantitative variables were expressed as mean ± standard deviation (SD), and qualitative variables were expressed as percent (%). Comparison of parametric values between four groups was performed using the one-way ANOVA test. Non-parametric values between four groups were analyzed using the Mann-Whitney U test. Categorical variables were compared by the Chi-square test or Fisher’s exact test. Stepwise, multivariate Cox regression analysis, which included variables with a P value of less than 0.1, was performed to identify independent predictors. Age, gender, history, duration of hospitalization, left ventricular EF, volume, CK-MB, potassium, percent of stenosis, diameter and length of the stent, HT, and admission Cr were included in the model. A two-sided P value of less than 0.05 was considered statistically significant. All statistical studies were carried out with SPSS program (version 15.0, SPSS, Chicago, IL, USA).

## Results

A total of 884 patients (113 female) undergoing primary PCI were enrolled to the study. Patients with renal dysfunction were often more female and older (P < 0.001 and P < 0.001) (Table 1). Even though patients with eGFR < 60 mL/min/1.73 m^2^ were more diabetic and hypertensive, this did not reach statistical significance (P = 0.137 and P = 0.072). The history of coronary artery disease and smoking did not differ between groups. With respect to laboratory findings, leucocyte count, CK-MB, admission glucose, and potassium levels were higher in the eGFR < 60 mL/min/1.73 m^2^ group (P = 0.004, P < 0.001, P < 0.001, and P < 0.001, respectively). The left ventricular EF significantly differed between groups (P < 0.001). Patients with eGFR < 60 mL/min/1.73 m^2^ group hospitalized longer (P = 0.004).

**Table 1:** Demographics of All Patients by Estimated Glomerular Filtration Rate

Demographics	Estimated glomerular filtration rates (mL/min/1.73 m^2^)				P
	< 60	≥ 60 - < 90	≥ 90 - 120	≥ 120	
Patient no., n (%)	72 (8.1)	390 (44.1)	345 (39)	77 (8.7)	
Gender (F/M), n	20/52	53/337	30/315	8/69	< 0.001
Age, years	59 ± 8.9	55.8 ± 8.3	51.1 ± 9	50.2 ± 8.5	< 0.001
Smoking, n (%)	38 (52.7)	238 (61.1)	211 (60.3)	57 (74)	0.118
CAD, n (%)	9 (12.5)	80 (20.5)	56 (16.2)	14 (18.2)	0.262
Diabetes mellitus, n (%)	30 (41)	113 (29)	66 (19)	19 (24.6)	0.137
Hypertension, n (%)	49 (68)	169 (43.3)	103 (29.8)	23 (29%)	0.072
Follow-up, months	18.9 ± 16	21.8 ± 15.6	22.8 ± 14.1	22.1 ± 13	0.245
Hospitalization, days	9.2 ± 5.3	7.8 ± 4.4	7.1 ± 3.8	7.7 ± 4.6	0.004
eGFR	50.5 ± 9.2	76.7 ± 7.3	101.3 ± 8	134 ± 12.2	< 0.001
Admission creatine, mg/dL	1.4 ± 0.4	1.03 ± 0.1	0.83 ± 0.07	0.67 ± 0.05	< 0.001
CK-MB, U/L	310 ± 215	216 ± 182	231 ± 177	225 ± 186	< 0.001
Leucocyte, 10^3^/µL	14.5 ± 4.7	13 ± 4	12.7 ± 3.4	12.6 ± 3.1	0.004
Hemoglobin, mg/dL	13.9 ±1.3	14.2 ± 1.3	14.1 ± 1.1	14.2 ± 0.9	0.625
Admission glucose, mg/dL	203 ± 104	161 ± 75	147 ± 64	157 ± 88	< 0.001
Potassium	4.4 ± 0.6	4.1 ± 0.5	4.1 ± 0.47	4.05 ± 0.49	< 0.001
Stent diameter, mm	3.1 ± 0.37	3.13 ± 0.36	3.15 ± 0.32	3.06 ± 0.34	0.277
Stent length, cm	21.4 ± 9	19.5 ± 6.8	18.5 ± 6.2	20.4 ± 5.8	0.013
Ejection fraction, %	42.2 ± 12	48.2 ± 10.2	49.2 ± 10.1	51.1 ± 8.8	< 0.001

Variables are reported as mean ± standard deviation or counts (percentages). F/M: female/male; CAD: coronary artery disease; eGFR: estimated glomerular filtration rate; CK-MB: creatine kinase-myocardial band.

In-hospital complications, including shock, ventricular arrhythmias, inotropic usage, atrial fibrillation, and intra-aortic balloon pump (IABP) usage were significantly different between groups (P < 0.001, P = 0.012, P < 0.001, P < 0.001, and P < 0.001, respectively) (Table 2). In-hospital MACE majorly driven by in-hospital mortality was significantly higher in eGFR < 60 mL/min/1.73 m^2^ group (P < 0.001 and P < 0.001, respectively). Even though the number was small, in-hospital stroke occurred more frequently in eGFR < 60 mL/min/1.73 m^2^ group (P = 0.07). Short- and long-term stent thrombosis and TVR were similar between groups (P = 0.133, P = 0.369, P = 0.464, and P = 0.247, respectively). Long-term MACE was higher in eGFR < 60 mL/min/1.73 m^2^ group and was driven by mortality and re-infarction (P = 0.028, P = 0.046, and P = 0.026, respectively).

**Table 2:** In-Hospital and Long-Term Events of All Patients by Glomerular Filtration Rate

	Estimated glomerular filtration rates (mL/min/1.73 m^2^)				P
	< 60	≥ 60 - < 90	≥ 90 - 120	≥ 120	
Shock, n (%)	8 (11.3)	13 (3.4)	4 (1.2)	-	< 0.001
Killip class > 1, n (%)	19 (34.5)	30 (11.1)	13 (5.1)	3 (5.5)	< 0.001
In-hospital mortality, n (%)	9 (12.5)	5 (1.3)	1 (0.3)	-	< 0.001
Re-infarction, n (%)	3 (4.2)	6 (1.5)	7 (2)	3 (3.9)	0.358
TVR, n (%)	5 (6.9)	19 (4.9)	12 (3.5)	5 (6.5)	0.464
MACE, n (%)	12 (16.7)	23 (5.9)	13 (3.8)	5 (6.5)	0.001
Stroke, n (%)	3 (4.2)	3 (0.8)	1 (0.3)	-	0.007
CPR, n (%)	10 (13.9)	9 (2.3)	5 (1.4)	-	< 0.001
Dialysis, n (%)	3 (4.2)	-	-	-	< 0.001
VT-VF, n (%)	9 (12.5)	15 (3.8)	14 (4.1)	3 (3.9)	0.012
CHD, n (%)	29 (40.3)	64 (16.4)	43 (12.5)	9 (11.7)	< 0.001
Inotrop usage, n (%)	19 (26.4)	37 (9.5)	29 (8.4)	2 (2.6)	< 0.001
IABP usage, n (%)	10 (13.9)	15 (3.8)	9 (2.6)	1 (1.3)	< 0.001
Atrial Fibrillation, n (%)	6 (8.3)	5 (1.3)	4 (1.2)	1 (1.3)	< 0.001
GIS bleeding, n (%)	1 (1.4)	2 (0.5)	2 (0.6)	-	0.723
Inguinal complication, n (%)	3 (4.2)	17 (4.4)	14 (4.1)	1 (1.3)	0.656
Thrombus (0 - 1 day), n (%)	1 (1.4)	1 (0.3)	3 (0.9)	1 (1.3)	0.533
Thrombus (1 - 30 days), n (%)	3 (4.2)	9 (2.3)	7 (2)	5 (6.5)	0.133
Thrombus (> 30 days), n (%)	3 (4.2)	8 (2.1)	8 (2.3)	-	0.369
Transfusion, n (%)	3 (4.2)	11 (2.8)	4 (1.2)	3 (3.9)	0.233
Cardiac death on follow-up, n (%)	6 (9.5)	12 (3.1)	11 (3.3)	1 (1.3)	0.046
CHD/Shock on follow-up, n (%)	13 (22.8)	38 (11.1)	25 (8.1)	5 (7.4)	0.008
Stroke on follow-up, n (%)	-	8 (2.4)	-	-	0.017
Re-infarction on follow-up, n (%)	10 (18.2)	28 (8.3)	21 (6.8)	9 (13.2)	0.026
TVR on follow-up, n (%)	18 (32.1)	76 (22.4)	63 (20.3)	17 (25)	0.247
MACE on follow-up, n (%)	24 (41.4)	95 (27.5)	71 (22.8)	20 (29.4)	0.028

Variables are reported as mean ± standard deviation or counts (percentages). Major adverse cardiac events included mortality, re-infarction and target vessel revascularization. TVR: target vessel revascularization; MACE: major adverse cardiac event; CPR: cardiopulmonary resuscitation; VT-VF: ventricular tachycardia-ventricular fibrillation; CHD: congestive heart disease; IABP: intra-aortic balloon pump; GIS: gastrointestinal system.

The unadjusted and adjusted models of logistic regression analysis for mortality according to eGFR are listed in Table 3. The mortality had the highest rates at eGFR < 60 mL/min/1.73 m^2^ level and had 7.78 times higher mortality rates (95% CI: 0.91 - 66.8) than eGFR > 120 mL/min/1.73 m^2^, which had the lowest rates as reference. After adjusted regression analysis, eGFR < 60 mL/min/1.73 m^2^ still had a 2.92 times higher mortality rate (95% CI: 0.23 - 36.8).

**Table 3:** Logistic Regression Models for In-Hospital Mortality by Estimated Glomerular Filtration Rate

Mortality	Estimated glomerular filtration rates (mL/min/1.73 m^2^)			
	< 60	≥ 60 - < 90	≥ 90 - 120	≥ 120
Model 1	7.78 (0.91 - 66.8)	2.41 (0.31 - 18.7)	2.49 (0.32 - 19.6)	1 (Reference)
Model 2	4.57 (0.5 - 41.1)	1.74 (0.21 - 13.9)	2.37 (0.29 - 18.7)	1 (Reference)
Model 3	4.3 (0.46 - 39.6)	1.81 (0.22 - 14.5)	2.49 (0.31 - 19.8)	1 (Reference)
Model 4	2.92 (0.23 - 36.8)	1.16 (0.12 - 10.8)	1.37 (0.14 - 12.7)	1 (Reference)

All data are presented as odds ratio (95% confidence interval). Logistic models: model 1: unadjusted model; model 2: adjusted model for age and sex; model 3: adjusted model for age, sex and past medical history; model 4: adjusted model for all covariates including age, gender, history, duration of hospitalization, left ventricular ejection fraction, CK-MB, potassium, diameter and length of the stent.

## Discussion

The present study demonstrated that any degree of renal impairment is associated with increased in-hospital and long-term mortality and MACE in patients presenting with STEMI undergoing primary PCI. Also, patients with renal impairment had lower left ventricular EF and higher cardiac enzyme. On the other hand, no relation was found between stent thrombosis and renal impairment for short- and long-term period.

Serum Cr level solely did not predict the severity of renal function. Even serum Cr level could stay in normal range until moderate and severe renal impairment developed [2, 7]. That is why eGFR was used for prediction of renal dysfunction. Generally, modification of diet in renal disease (MDRD) formula was used for prediction of eGFR in previous studies and in clinical practice [2, 7]. In our previous study, we found that C-G derived eGFR predicts worse prognosis better than MDRD in STEMI [19]. This was a major difference between present and previous studies.

It was demonstrated that renal impairment is more frequently associated with HT and DM in addition to older age and female gender [7]. These clinical factors are associated with poor prognosis in acute MI [7]. Even though in the present study patients with renal impairment are mostly female, older and more frequently had HT and DM, after multivariate regression analysis, eGFR derived renal impairment is still prognostic in STEMI patients undergoing primary PCI.

The relation between CKD and cardiovascular mortality is well known [1-3]. Several different studies have shown that even mild degree of renal impairment is related with increased adverse outcomes in normal population and patients with cardiac disease. A similarly designed study with the present one shows that an eGFR < 30 mL/min/1.73 m^2^ is associated with increased in-hospital and 1-year mortality [20]. In the present study, we show that long-term mortality and adverse events are sustained beyond 2 years. And also regression analysis showed that mild degree of renal impairment is related with adverse events. There are some potential explanations why renal impairment is associated with worse outcomes. Firstly, it has shown that as eGFR decreased, fibroblast and parathyroid hormone levels increased. As a result, vitamin D and phosphate metabolisms are deteriorated which leads to vascular dysfunction and coronary artery disease [6, 21, 22]. Secondly, potential explanation is that renal impairment is associated with enhanced pro-coagulant state, increased homocysteine levels, and endothelial dysfunction that eventually lead to atherosclerotic plaque formation and destabilization [2, 23]. A recent study by Duran et al showed that mild and moderate renal impairment is associated with increased atherosclerosis burden [13].

Another important finding of our study was that as left ventricular EF decreased, eGFR also decreased. Also, CK-MB level was highest in the eGFR < 60 mL/min/1.73 m^2^ group. In a recent study, Sonmez et al found that in patients with STEMI undergoing primary PCI, an eGFR < 60 mL/min/1.73 m^2^ is associated with lower left ventricular EF and larger myocardial infarct size [12].

Some other findings of the present study were in accordance with the previous studies. The hospitalization duration was longer in lowest eGFR group as in Alfaleh et al’s study, probably as a result of increased in-hospital adverse events [24]. Previous reports indicated that the risk of stroke increases with renal impairment [24, 25]. Even though the number of stroke was low, in the present study, there was a significant inverse relation with eGFR and stroke.

In the present study, the rate of stent thrombosis did not differ significantly between groups. There are different results in previous studies in regard of different types of stents. In a study performed by Miao et al, CKD, in elective DES implantation patients, was associated with increased stent thrombosis [26]. Another study that excluded STEMI patients showed that in patients with eGFR < 60 mL/min/1.73 m^2^, DES usage is not associated with stent thrombosis but reduced myocardial infarction and rate of repeat revascularization [27]. Similar to Wang et al’s study, Toutouzas et al showed that new generation DES did not increase the rate of stent thrombosis in CKD patients [28]. Another study performed with bare metal stents showed an inverse relation between renal dysfunction and neo-intima formation after BMS deployment. In that study the neo-intima intravascular ultrasound showed greater lipid volume, suggesting atherosclerosis development [29]. In the present study, BMS was deployed for stent implantation. For BMS, restenosis rather than thrombosis is a concern [30]. Probably this could be the explanation why no difference of stent thrombosis was found between groups.

The present study had several limitations. Firstly, this study was retrospective and single-centered. Secondly, just the effect of admission Cr derived eGFR was used for assessment. Thirdly, the impact of in-hospital medication on prognosis and eGFR was not assessed. Fourthly, the standard method of renal function assessment, 24 h Cr clearance, was not performed.

### Conclusions

In conclusion, the present study demonstrated that any degree of renal impairment and especially eGFR < 60 mL/min/1.73 m^2^ was associated with increased in-hospital and long-term mortality and MACE and decreased left ventricular EF. C-G derived eGFR is a simple, easily bedside applicable method of renal impairment for risk stratification in STEMI patients undergoing primary PCI.
